# Calcium Increase in Blood

**Published:** 1909-01-30

**Authors:** 


### CALCIUM INCREASE IN BLOOD.

There have been considerable differences of opinion as to the extent to which the percentage of calcium salts in the blood can be increased by the therapeutic administration of calcium compounds by the mouth. One of the chief causes why the question is so difficult of answer is that there is no easy method of estimating calcium with any degree of accuracy in the comparatively small quantities of blood that are obtainable from patients. The experimental error is so great that hardly any conclusions can be drawn. Dr. T. R. Boggs, working in the Clinical Laboratory of the Johns Hopkins Hospital, took a great deal of pains to perfect a method of calcium estimation—we need not give the details of it here—and then used it in analysing the blood of dogs before and after the administration of calcium salts and other remedies. He concludes that beyond doubt the calcium content of the blood in normal dogs may be very greatly increased by feeding on calcium salts, and that this increase is maintained several days. He thinks it is probable that small therapeutic doses of certain simple fatty acids diminish the calcium content of the blood, though to a less degree. The importance of this in practical medicine is obvious; the most common use of calcium as a medicine is in cases—such as purpura, urticaria, aneurysm—in which it is desired to raise the calcium content of the blood, and thus increase the coagulability of the latter. The whole basis of calcium therapeutics would crumble if it were proved that it is not possible to alter the percentage of it in the blood by administering it orally; apparently, however, it can be altered. The next thing we require to know is exactly how high a figure it is likely to reach upon a given dosage of calcium by the mouth, because up to a certain point increasing the calcium in the blood increases the coagulability of the latter, but a further increase in the calcium beyond that point has exactly the reverse action, diminishing the coagulability of the blood again as much as if the percentage of calcium were below normal. But the conclusions of Dr. Addis, summarised in our last issue, must be carefully borne in mind in this connection.

